# The PCSK9 discovery, an inactive protease with varied functions in hypercholesterolemia, viral infections, and cancer

**DOI:** 10.1016/j.jlr.2021.100130

**Published:** 2021-10-02

**Authors:** Nabil G. Seidah

**Affiliations:** Laboratory of Biochemical Neuroendocrinology, Montreal Clinical Research Institute (IRCM, Affiliated to the University of Montreal), Montreal, Quebec, Canada

**Keywords:** proprotein convertases, furin, lipid metabolism, LDL-C, LDLR, lysosomes, hypercholesterolemia, inflammation, viral infection, cancer/metastasis, cDNA, complementary DNA, CHRD, Cys-His-rich domain, DENV, dengue virus, ER, endoplasmic reticulum, FH, familial hypercholesterolemia, GOF, gain-of-function, HEK293, human embryonic kidney 293, LDLR, LDL receptor, LOF, loss of function, mAb, monoclonal antibody, MHC, major histocompatibility complex, NARC-1, neural apoptosis-regulated convertase 1, PC, proprotein convertase, PCSK9, proprotein convertase subtilisin/kexin type 9, S1P, site 1 protease, SKI-1, subtilisin-kexin isozyme 1

## Abstract

In 2003, the sequences of mammalian proprotein convertase subtilisin/kexin type 9 (PCSK9) were reported. Radiolabeling pulse-chase analyses demonstrated that PCSK9 was synthesized as a precursor (proPCSK9) that undergoes autocatalytic cleavage in the endoplasmic reticulum into PCSK9, which is then secreted as an inactive enzyme in complex with its inhibitory prodomain. Its high mRNA expression in liver hepatocytes and its gene localization on chromosome 1p32, a third locus associated with familial hypercholesterolemia, other than *LDLR* or *APOB*, led us to identify three patient families expressing the PCSK9 variants S127R or F216L. Although *Pcsk9* and *Ldlr* were downregulated in mice that were fed a cholesterol-rich diet, PCSK9 overexpression led to the degradation of the LDLR. This led to the demonstration that gain-of-function and loss-of-function variations in PCSK9 modulate its bioactivity, whereby PCSK9 binds the LDLR in a nonenzymatic fashion to induce its degradation in endosomes/lysosomes. PCSK9 was also shown to play major roles in targeting other receptors for degradation, thereby regulating various processes, including hypercholesterolemia and associated atherosclerosis, vascular inflammation, viral infections, and immune checkpoint regulation in cancer. Injectable PCSK9 monoclonal antibody or siRNA is currently used in clinics worldwide to treat hypercholesterolemia and could be combined with current therapies in cancer/metastasis. In this review, we present the critical information that led to the discovery of PCSK9 and its implication in LDL-C metabolism. We further analyze the underlying functional mechanism(s) in the regulation of LDL-C, as well as the evolving novel roles of PCSK9 in both health and disease states.

In the last 30 years, treatment of patients suffering from familial hypercholesterolemia (FH) was limited to the use of “statins” to inhibit cholesterol synthesis, and later, ezetimibe was added to block the absorption of cholesterol by the gut. In 2003, the discovery of proprotein convertase subtilisin/kexin type 9 (PCSK9) provided an extraordinary paradigm shift, as it led to the development of a very powerful arsenal of inhibitory agents that effectively lower LDL-C to unprecedented low levels. Since 2016, subcutaneous injections of monoclonal antibodies (mAbs) targeted against circulating PCSK9 effectively blocked its ability to enhance LDL receptor (LDLR) degradation and hence very significantly lowered LDL-C levels way above the effect of statins. These are now prescribed in more than 30 countries. Very recently, subcutaneous injection of antisense siRNAs against PCSK9 targeted to liver led to a more convenient approach requiring twice a year administration. The present review provides a historical perspective of the discovery of PCSK9 and the clinical applications of its inhibitors for LDL-C lowering and beyond.

## Limited proteolysis of secretory proteins

Endocrine and exocrine secretory proteins are first synthesized in the endoplasmic reticulum (ER), and upon exit from this compartment, they reach their destinations inside the cell, at the cell surface, or are secreted. These include polypeptide hormones, receptors, enzymes, growth factors, and even toxins and viral glycoproteins. During their cellular transit, they undergo a variety of post-translational modifications, including N-glycosylation and O-glycosylation, Ser/Thr-phosphorylation and Tyr-phosphorylation, Tyr-sulfation, Cys-palmitoylation, Ser-octanoylation, N-acetylation, C-terminal amidation, as well as irreversible peptide bond cleavages. Such post-translational modifications affect the biological functions and/or half life of secretory proteins, thereby expanding by more than a thousandfold the diversity of the proteins encoded by the genome. Based on the human protein atlas (https://www.proteinatlas.org/humanproteome/tissue/secretome), approximately 7,300 of the 19,670 genes encode one or more secreted protein (∼1,770) or proteins containing at least one transmembrane domain (∼5,530). During embryonic development and throughout adult life, the irreversible limited proteolysis of secreted precursor proteins and their polypeptide derivatives generates new sets of products with distinct biological functions and fates (1). Limited proteolysis essentially occurs at specific single or pairs of basic amino acids to release bioactive proteins or peptides (2, 3, 4). Enormous, collective, and sustained efforts from research groups revealed that limited proteolysis of secretory proteins occurs in the trans-Golgi network, immature secretory granules, cell surface and endosomes following cleavage at the general motif (**K**/**R**)-Xn-(K/**R**)↓, where Xn represents a spacer sequence composed of 0, 2, 4, or 6 amino acids (2, 5). Note that Arg is largely preferred over Lys at the P1 position, just before the cleavage site, and is preceded by one or more basic residue(s) at positions P2, P4, and/or P6 (6).

## The basic amino acid-specific proprotein convertases

The wide variety of secretory precursor proteins that underwent cleavages to release their bioactive segments or even to generate novel functions distinct from parent proteins/peptides (7) led to intense efforts to identify the cognate proteases during the period of 1967–1989. This proved to be a very arduous and formidable task, with many attempts ending up in failure because of the scarcity of the processing enzymes compared with their substrates and the lack of sensitive and specific assays (8). Major advances in molecular and cell biology, including the power of yeast genetics, allowed the identification in 1984 of Kex2p in *Saccharomyces cerevisiae*, now called kexin, as the processing enzyme of the hormonal α-mating factor precursor at four **KR**↓EA sites (9). In 1988, the analysis of the nucleotide sequence coding for kexin revealed that this prototype of the eukaryotic proprotein convertases (PCs) was homologous to bacterial subtilisin, a serine protease that comprises a typical Asp∗, His∗, and Ser∗ catalytic triad and an Asn∗ at the oxyanion hole (10). Thus, the yet to be discovered mammalian PCs could be homologous to ancient subtilases rather than to the more recent family of trypsin/chymotrypsin-like serine proteases (11). The same year, attempts to identify the convertases of proinsulin in human insulinoma lysates led to the identification of two calcium-dependent acidic proteases, type-I and type-II endopeptidases, sequentially required to generate bioactive insulin in secretory granules (12).

**Fig. 1:**
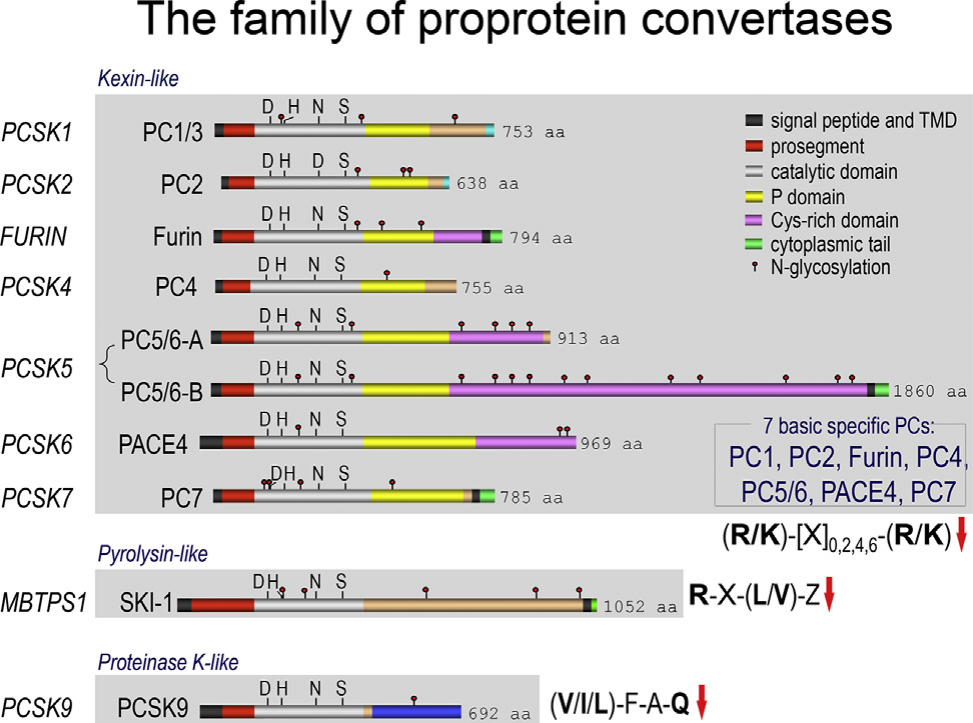
Schematic representation of the primary structures of the family of proprotein convertases. The kexin-like basic amino acid-specific PCs, pyrolysin-like SKI-1/S1P and proteinase K-like PCSK9 are separated to emphasize their distinct subclasses. The various domains and N-glycosylation positions are emphasized. Notice that only PC5/PC6 exhibits two validated alternatively spliced forms, namely PC5/PC6A and PC5/PC6B. The 4 membrane-bound human PCs include furin, PC5/PC6B, PC7, and SKI-1/S1P. The presence of a signal peptide, a prosegment, and catalytic domain is common to all convertases that exhibit the typical catalytic triad residues Asp, His, and Ser and the oxyanion hole Asn (Asp for PC2). Following the catalytic domain, all the basic amino acid-specific convertases, except for SKI-1/S1P and PCSK9, exhibit a P domain that apparently stabilizes the catalytic pocket. The C-terminal domain of each convertase contains unique sequences regulating their cellular localization and trafficking. PC5/PC6 and PACE4 contain a specific Cys-rich domain (CRD). In contrast, PCSK9 exhibits a C-terminal CHRD that is required for the trafficking of the PCSK9-LDLR complex to endosomes/lysosomes.

The sequence and molecular identification in cells and tissues of the first two mammalian PCs (Fig. 1), namely PC1 (13, 14) and PC2 (13, 15), was finally achieved in 1990 following PCR amplifications of subtilase-like and kexin-like transcripts using degenerate oligonucleotides. Their genes were designated *PCSK1* and *PCSK2* to refer to their homology to subtilisin and kexin (8). PC1 and PC2 turned out to be the major endocrine and neural PCs that are implicated in the processing of the vast majority of polypeptide hormones in the regulated secretory pathway, resulting in the formation of bioactive hormones stored in dense core secretory granules, for example, insulin, adrenocorticotrophic hormone, β-endorphin, α-melanotropin, enkephalins, glucagons, and many others (8). Indeed, major endocrinopathies, such as obesity, diabetes, and gastrointestinal disorders, implicate the loss of function (LOF) of PC1 (16, 17, 18, 19, 20).

Independently, a third PC, furin (gene *FURIN*; *PCSK3*), was identified in 1989–1990 (21, 22). This ubiquitously expressed type-I membrane-bound subtilase was found to cycle between the trans-Golgi network, cell surface, and endosomes (5) and is the major convertase of the constitutive secretory pathway implicated in the activation of a large variety of soluble and membrane-bound proteins, including growth factors, enzymes, receptors, toxins (5, 23, 24), and surface glycoproteins of infectious enveloped viruses, such as HIV-gp160 and the spike glycoprotein of severe acute respiratory syndrome coronavirus 2 (25, 26).

Between 1992 and 1997, four other PCs exerting a wide variety of functions were identified, namely the soluble gonad-specific PC4 (*PCSK4*), the widely expressed PC5 (*PCSK5*) and PACE4 (*PCSK6*), and the type-I membrane bound PC7 (*PCSK7*) (8). Some of these enzymes, as well as furin, can not only activate proteins (27, 28) but also contribute to their inactivation (7), as we shall see later for the furin-mediated inactivation of PCSK9 (29, 30). In some cases, PCs may share the same substrates (redundancy).

To conclude, the first seven PCs (Fig. 1) cleave their substrates after one or more basic residues and play mostly specific but sometimes redundant roles. They fulfill key functions during embryonic development, several of them being essential (31, 32, 33), as well as in the adult and under certain pathological conditions (7, 8).

## The nonbasic residue-specific PC subtilisin-kexin isozyme 1/site 1 protease

In a variation on the theme of secretory precursor processing, it became apparent that some proteins are cleaved at nonbasic amino acids along the secretory pathway in the *cis/medial* Golgi (34). This unexpected twist of events led us to study in detail the processing of the precursor of brain-derived neurotrophic factor (pro-brain-derived neurotrophic factor). Although it was already known that bioactive brain-derived neurotrophic factor (amino acids 129–247) production required cleavage at the **R**V**RR**_128_↓HS site, we unexpectedly noticed that another earlier processing event occurred at **R**G**L**T_57_↓SL, exhibiting a critical Arg and Leu at the P4 and P2 positions, respectively (35), a general motif later shown to be **R**-X-**aliphatic**-Z↓, where X and Z are variable amino acids (25, 36). This observation led us to identify and clone the complementary DNA (cDNA) of the cognate type-I membrane-bound subtilase homologous to the bacterial pyrolysin, the eighth member of the PC family (Fig. 1), which we called subtilisin-kexin isozyme 1 (SKI-1) (35). Independently, the same protease was found to process membrane-bound transcription factors, such as SREBPs 1 and 2, and was called “site 1 protease” (S1P) (37) and its gene “membrane-bound transcription factor protease site-1.” Thus, SKI-1/S1P regulates important physiological functions such as cholesterol and fatty acid metabolism (37), ER stress (38), bone metabolism (39), as well as the phosphorylation of mannose residues of proteins destined for lysosomal sorting (40). It was also realized that certain infectious hemorrhagic fever viruses exploit this mechanism to activate their surface glycoprotein and enhance their cellular infection and entry into various tissues (25).

## The discovery of PCSK9 and its regulation of LDL-C

Because the human genome was not yet completed, we hypothesized that other members of the PC family homologous to SKI-1/S1P may be yet to be discovered (11). Here again, we used PCR to amplify cDNAs coding for mRNAs homologous to that of SKI-1/S1P. Using degenerate oligonucleotides that hybridize with the conserved DNA sequences surrounding the codons of the active sites Ser∗ and His∗ of SKI-1/S1P led us to clone the cDNA and analyze the biosynthesis of the ninth and last member of the PC family. Upon screening databases for similar sequences, we noticed that Millennium Pharmaceuticals had released a partial cDNA in a patent database as belonging to a group of genes upregulated upon induction of apoptosis in primary cerebellar neurons by serum withdrawal (patent no.: WO 01/57081 A2). Because of its modulation in conditions leading to apoptosis, they named the encoded protein neural apoptosis-regulated convertase 1 (NARC-1). Similarly, *via* global cloning of secretory proteins, Eli Lilly had identified a partial sequence called LP251 (patent no.: WO 02/14358 A2). Upon completion of the human, mouse, and rat sequences of this novel subtilase, we kept the NARC-1 acronym but substituted the word “candidate” by “convertase” (41). However, in the same year, the editor of *Nature Genetics* suggested that we rename the NARC-1 protein and gene PCSK9 and *PCSK9*, respectively, based on the recommendation of Human Gene Nomenclature Committee (https://www.genenames.org/) (42). Interestingly, “*K*” in the gene name of the first seven PCs evokes their homology to the kexin subfamily of subtilases, whereas “*K*” in PCSK9 also underlines its similarity to the proteinase K subfamily of subtilases (41). PCSK9 catalytic domain exhibits 25% of sequence identity to that of its closest family member SKI-1/S1P (41). The human PCSK9 mRNA (NM_174936.3) spans 3,710 bp over 12 exons encoding a 692 amino-acid protein (NP_777596.2).

**Fig. 2:**
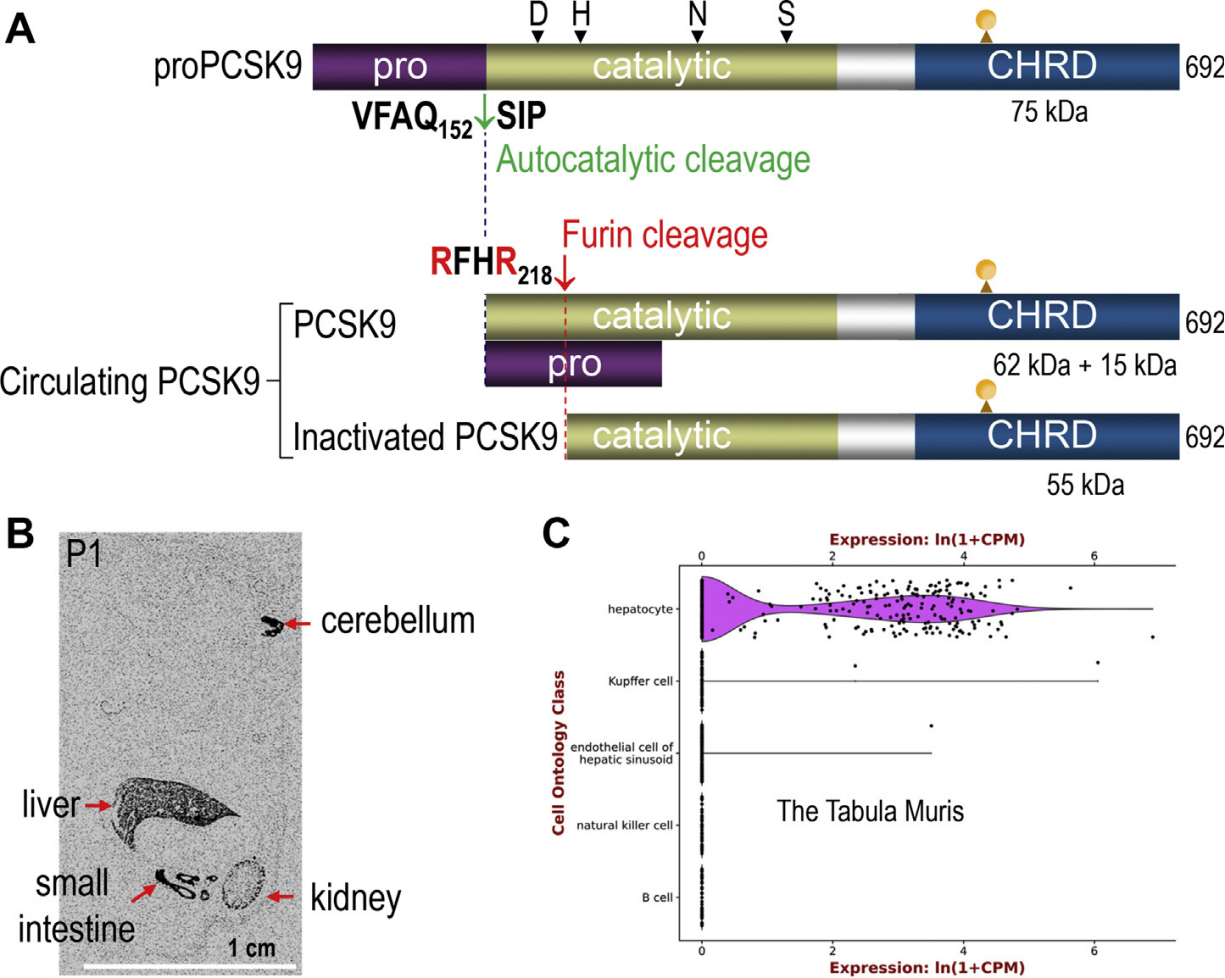
Schematic overview of the biosynthesis of PCSK9, its furin cleavage, and tissue expression. A: The ∼75 kDa precursor of PCSK9 (proPCSK9) is autocatalytically cleaved at **V**FA**Q**_152_↓ in the ER to excise the inhibitory ∼15 kDa prodomain that remains noncovalently attached to the ∼62 kDa catalytic subunit of PCSK9. The complex is thus secreted as an inactive protease, which can enhance the degradation of the LDLR. At the cell surface, the convertase furin cleaves a fraction of the complex at **R**FH**R**_218_↓ to release a C-terminal ∼55 kDa product that has no activity on the LDLR (LOF). The positions of the active sites Asp∗, His∗, Ser∗, and the oxyanion hole Asn∗ are shown, as well as that of the single N-glycosylation site (CHO). B: Whole mount in situ hybridization histochemistry of the mRNA expression of mouse PCSK9 at post-partum day 1 (P1). The liver exhibits the highest expression of PCSK9 followed by the small intestine, kidney, and cerebellum. The scale bar of 1 cm is shown. C: A Tabula Muris Consortium that cumulated hundreds of liver single-cell transcriptomics by RNA-Seq (czbiohub.org) revealed that PCSK9 transcripts are exclusively expressed in hepatocytes.

Since we did not have an antibody to PCSK9 yet, we inserted a V5 tag at the C terminus of the protein and analyzed its protein expression and maturation following transient transfection of its cDNA in human embryonic kidney 293 (HEK293) cells. Pulse labeling of the cells expressing human or mouse PCSK9-V5, or its active site H226A mutant, with ^35^S-Cys/Met for 10 or 20 min was followed by chase times of 30, 60, and 120 min in the absence of radiolabel. Immunoprecipitations of cell lysates and media with a V5-mAb was followed by separation of the immunoprecipitated proteins on SDS-PAGE. Like all other PCs, the data showed that when the active site His∗ was mutated to Ala, the ∼75 kDa proPCSK9 (Fig. 2A) was not autocatalytically cleaved, remained in the ER (except for PC2), and was not secreted. In contrast, native PCSK9 was autocatalytically cleaved in the ER (41) at **V**FA**Q**_152_↓ (43, 44), resulting in a prodomain-PCSK9 complex (Fig. 2A) that was secreted into the medium. The processing at P1 Gln_152_↓ was found to be sensitive to the presence of Val at P4 (45). Thus, different from all eight other PCs, only for PCSK9, does the ∼15 kDa inhibitory prodomain (46) remains noncovalently associated with the mature ∼62 kDa catalytic subunit (Fig. 2A), even when secreted (41).

In situ hybridization (Fig. 2B), tissue/cell line analyses by Northern blotting, and RT-quantitative PCR revealed that liver, small intestine, and pancreatic β-cells (βTC3 and Rin-m5F) are the major sources of PCSK9 in adult mouse and rat (41, 47). In the liver, PCSK9 expression is confined to hepatocytes, as its transcripts were undetectable in hepatocyte-specific PCSK9 KO liver (48) and were only detected in hepatocytes by liver single-cell transcriptomics (49) (Fig. 2C). During embryonic development, PCSK9 transcripts are transiently detected by *in situ* hybridization in the brain telencephalon and cerebellum (Fig. 2B) (41). This tissue distribution of PCSK9 was unique with respect to that of other convertases. Its remarkably high expression in hepatocytes suggested that it was implicated in the regulation of metabolic functions. To probe the latter, liver partial hepatectomy was performed in rat, and PCSK9 expression was monitored during regeneration. PCSK9 mRNA increased by ∼2.5-fold 48 h post-liver partial hepatectomy, suggesting that PCSK9 is implicated in hepatocyte proliferation. This report first appeared in February 2003 (41).

**Fig. 3:**
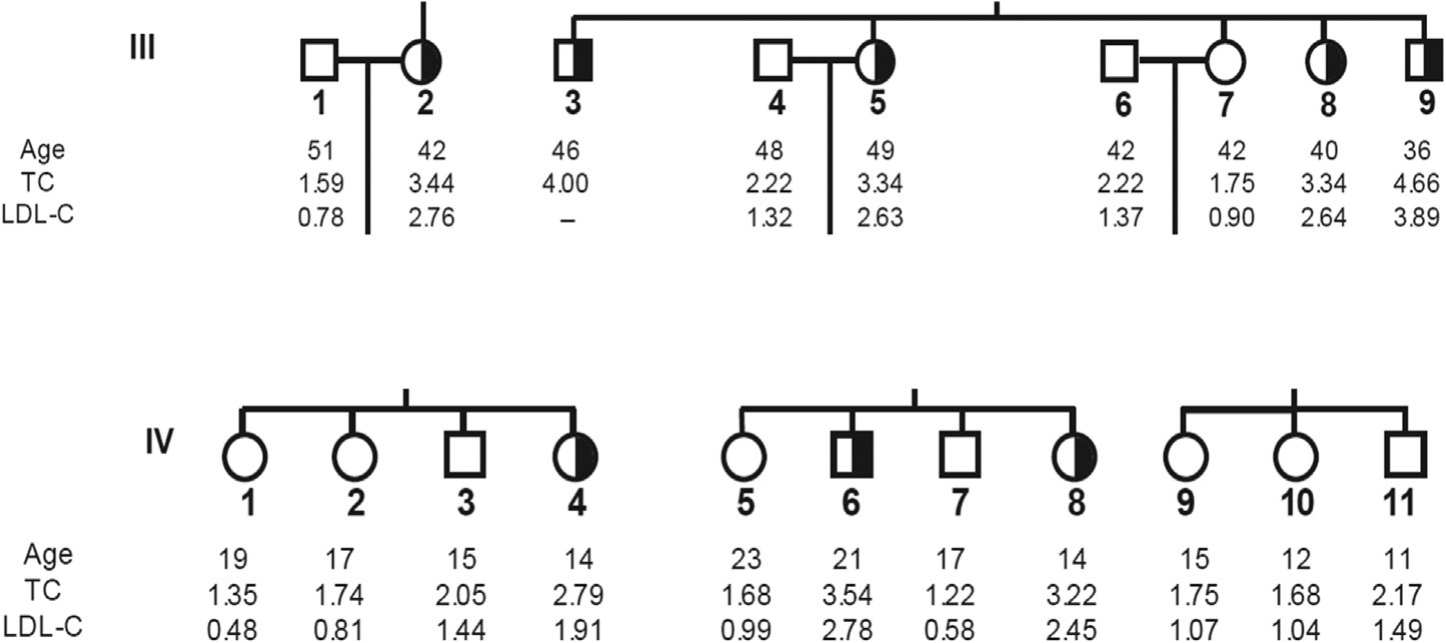
Partial pedigree of FH individuals exhibiting the variant S127R in families III and IV and their direct siblings. Black fillings indicate the mutated heterozygote allele in both males and females. Age (in years) at lipid measurement, total cholesterol (TC), and LDL-C (in grams per liter) are given. Much more detailed data and the pedigree of the family presenting the F216L genotype can be found in the original article by Abifadel *et al.* (42) that was published in June 2003.

Stunningly, upon analyzing the *PCSK9* location on chromosome 1p32 (41), we realized that it was close to a locus, 1p34.1-p32, possibly encoding a third gene implicated in autosomal dominant FH. The latter was identified in 1999 in two large French families, in which the *LDLR* and *APOB* genes were excluded (50). Moreover, a similar study including a very large Utah family affected by severe hypercholesterolemia also circumscribed it to chromosome 1p32 (51). Armed with this information and our demonstration of a major expression of PCSK9 in liver and small intestine (41), both of which are important in cholesterol synthesis and regulation, we initiated an active collaboration with the French team led by Catherine Boileau. Sequencing of the 22-kb *PCSK9* gene in all their patients, which had not yet been performed, led to the identification of the missense variants S127R (exon 2) (Fig. 3) and F216L (exon 4). This was reported by Abifadel *et al.* (42) in June 2003. The data clearly demonstrated that these dominant heterozygote missense variations in PCSK9 are associated with a substantial 1.5- to 3.5-fold elevation in circulating LDL-C levels (Fig. 3) (42), suggesting a gain-of-function (GOF) or a dominant negative mechanism. In 2004, the D374Y variant that resulted in even higher levels of circulating LDL-C was simultaneously identified in a Utah pedigree (52) and Norwegian subjects (53).

## PCSK9 protein enhances the degradation of the LDLR in endosomes/lysosomes

The first mechanistic explanation of how PCSK9 regulates LDL-C levels was the demonstration by Maxwell and Breslow in 2004 that adenoviral overexpression of PCSK9 severely reduced the LDLR protein but not its mRNA levels in the hepatocyte-derived cell line McARH7777 and in mice, by inducing its degradation (54, 55) within the acidic endosomal/lysosomal pathway (44, 55, 56). The same authors had shown a few months earlier by microarray analysis that *PCSK9* was strongly downregulated by dietary cholesterol, providing evidence that *PCSK9* is a cholesterol-regulated gene (57). This conclusion was supported by a similar study implicating SREBP1-a or SREBP-2 overexpression in mice that led to higher levels of PCSK9 (58) and by the ability of statins to enhance *PCSK9* transcription (59). Thus, PCSK9 and LDLR mRNA levels were both positively regulated by the lack of cholesterol and by statin treatment, but PCSK9 induced LDLR protein degradation (60).

Coming from the PC side of this story, it was difficult to admit that, except toward itself, PCSK9 had no *in trans* protease activity. In addition, it was highly unusual to associate autosomal dominant mutations to a gene encoding an active protease. Indeed, how could several point mutations involving distinct single amino acid substitutions result in a gain of enzymic activity? We performed many assays, in which we preincubated the enzyme in all sorts of buffers to possibly trigger the separation of the inhibitory prodomain from the enzyme. In all cases, removal of the prodomain invariably led to PCSK9 aggregation, as also observed later in another study (61). This led to us to conclude that the association of the prodomain with PCSK9 likely results in a stable bioactive complex. Since the prodomain of PCs is usually a strong inhibitor of the parent protease (46, 62), it was therefore not unlikely that the secreted prodomain-PCSK9 complex is enzymatically dead, and that it may exert its functions by a nonenzymatic mechanism.

In 2005, Cohen *et al.* (63) showed that subjects from the Dallas Heart Study exhibiting substantial (∼40%) reductions in LDL-C levels carried truncating Y142X or C679X heterozygote LOF mutations. The fact that heterozygote mutations were associated with a severe drop in LDL-C was the first strong evidence that PCSK9 may be acting stoichiometrically on the LDLR, rather than as a protease. Usually for a catalytically active enzyme, a substantial loss of activity with little residual function would be needed to have a noticeable effect on its bioactivity. For example, it was reported that in mice it took a conditional liver-specific knockout of SKI-1/S1P of more ≥95% to have a functional defect in activated SREBPs (64). As a corollary, PCSK9 GOF variants such as S127R, F216L (42), and D374Y (52, 53), which increased the ability of PCSK9 to further promote LDLR degradation, most likely result in an enhanced nonenzymatic activity. In the same year, *Pcsk9* inactivation in mouse confirmed that the loss of PCSK9 expression was associated with ∼3-fold higher LDLR levels in the liver and with a dramatic reduction of plasma LDL-C (65). A similar observation was also obtained in complete and hepatocyte-specific PCSK9 KO mice (48). The viability of these KO mice (48, 65) as well as the discovery in 2006 and 2007 of the first seemingly healthy individuals completely lacking functional PCSK9 (66, 67) firmly established PCSK9 as an attractive therapeutic target for LDL-C reduction.

Interestingly, in our first report, we noticed that secreted PCSK9 was partially cleaved into a ∼8 kDa shorter product by an endogenous protease in HEK293 cells (41). We investigated this product further and found it to result from a furin cleavage of mature PCSK9 at **R**FH**R**_218_↓ (29). This led to the separation of the prodomain and the N-terminal segment 153–218 from the main body of the protein, resulting in a ∼55 kDa PCSK9 C-terminal fragment comprising amino acids 219–692 (Fig. 2A) that was inactive on the LDLR (29). This PCSK9-inactivating role of cell-surface furin was confirmed in mice lacking furin in hepatocytes (30) and shown to occur in humans, as the truncated PCSK9 that has no activity on the LDLR represents ∼30% of total circulating PCSK9 (68). Thus, we propose that the three PCSK9 GOF variants F216L (42), R218S (69), and R215H (70) are due to the loss of the furin cleavage at this site, resulting in higher levels of bioactive PCSK9.

In contrast, the mechanism underlying the GOF of the variant PCSK9-S127R resulting in enhanced circulating LDL-C (42) is still not clear. Kinetic studies in cells, mice, and human patients suggested that this variant enhanced the production levels of apoB containing VLDL particles (71, 72). It was thus concluded that the effect of the S127R mutation of PCSK9 on plasma cholesterol homeostasis is mainly related to an overproduction of apoB-100. Yet, biosynthetic analyses of this variant clearly showed that the zymogen processing of proPCSK9 and secretion of mature PCSK9 were significantly lower, with higher levels of unprocessed ER-retained proPCSK9 relative to the mature PCSK9 (44, 56). Could it be that within the ER, at higher levels, proPCSK9-S127R may act as a chaperone protecting ApoB from degradation and hence resulting in higher ApoB secretion? Alternatively, the mature PCSK9-S127R variant may have a more active intracellular activity (73) on the LDLR (74), possibly leading to increased apoB secretion. Indeed, it was reported that LDLR-null homozygote FH patients exhibit a ∼2-fold higher apoB-100 production rate (75, 76). In addition, incubation of HEK293 or HepG2 cells with PCSK9-S127R *versus* WT PCSK9 resulted in a lower LDL uptake, in accord with a GOF on the LDLR (77). The fact that mature PCSK9-S127R has an increased affinity for heparin-like coreceptor molecules and binds more strongly to the LDLR (74) also support a GOF phenotype (78). Altogether, the S127R variation may have multiple GOF consequences that ultimately lead to higher circulating LDL-C.

In 2007, it was shown that PCSK9 directly binds the epidermal growth factor precursor homology domain A of the LDLR (79). Simultaneously, *in vitro* binding assays demonstrated that, at neutral pH, PCSK9 D374Y binds the LDLR with a ∼35-fold higher affinity (61), thereby rationalizing the dramatic phenotype of this GOF variant (52). The 3D structure of circulating PCSK9 secreted from liver (80) showed that the C terminus of the prodomain was solidly embedded in the substrate-binding groove (61, 81), likely blocking access to any substrate. These structural data confirmed our original observation of the secretion of mature PCSK9 as a noncovalent complex with its inhibitory prodomain (41).

In agreement, coexpression of the prodomain of PCSK9 with a catalytically dead mutant of mature PCSK9 in which the active site Ser_386_ was mutated to Ala (S386A) led to a reconstituted fully functional and secreted PCSK9 that can mediate LDLR degradation, similar to WT PCSK9 (82). Interestingly, when a similar experiment was attempted with yeast kexin, a functional protease was also produced, consistent with a model whereby covalent linkage to the protein is not an absolute requirement for a PC prodomain to function as an intramolecular chaperone and inhibitor (83). This conclusion was later confirmed in another study using a similar approach with a PCSK9 mutant of the active site His_226_ (H226A) that also resulted in the enhanced degradation of other LDLR-family members, namely VLDL receptor and ApoER2 (84). Thus, different from the first eight convertases (8), PCSK9 exhibits a protease activity only once during its autocatalytic zymogen processing in the ER, and then acts in *trans* on target proteins, such as cell surface receptors, by escorting them to endosomes/lysosomes for degradation (85).

**Fig. 4:**
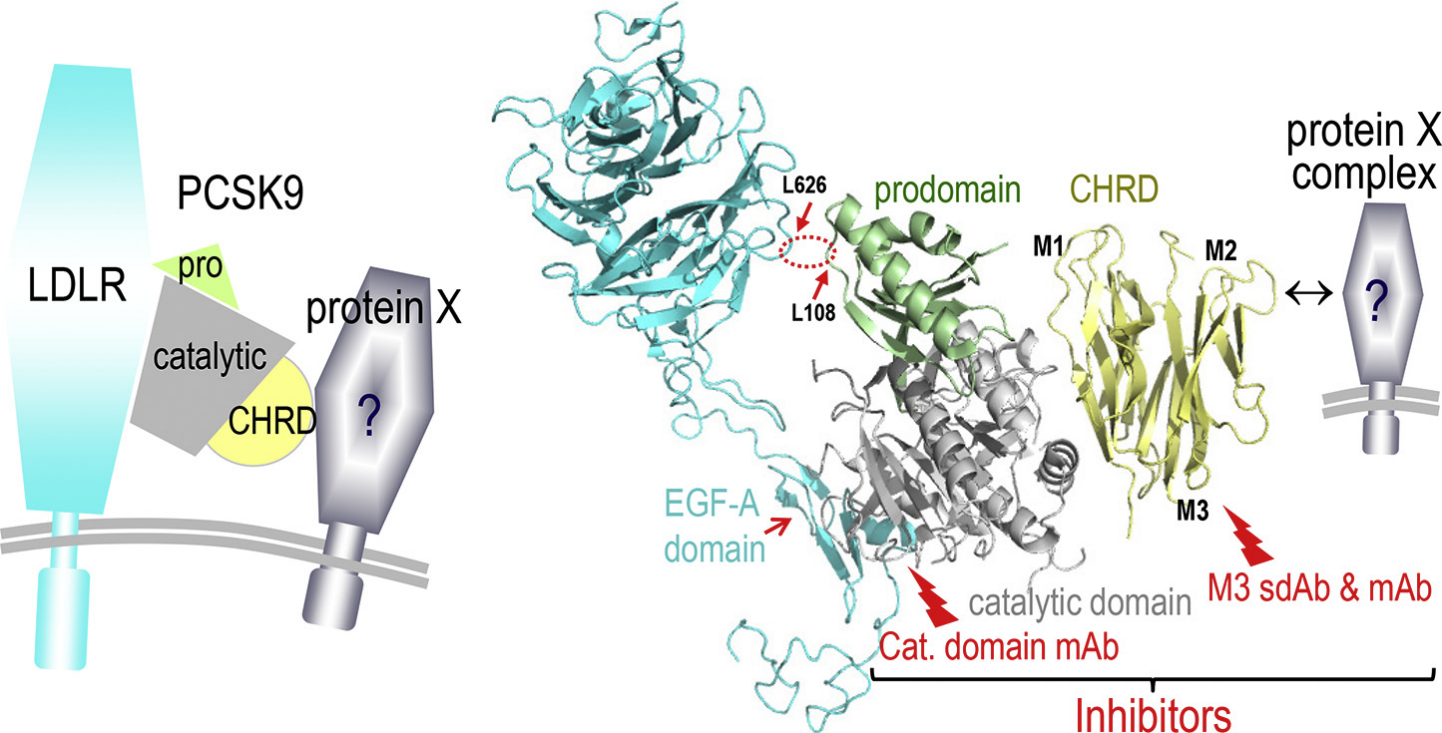
Schematic representation of the PCSK-LDLR complex and a modified version of a cocrystal structure representing the binding of the catalytic domain of PCSK9 to the epidermal growth factor precursor homology domain A of the LDLR, as well as the hydrophobic interaction of Leu_108_ of the prodomain of PCSK9 with Leu_626_ in the β-barrel domain of the LDLR (dashed red ellipse) (87). The inhibitory role of the widely prescribed mAbs against the catalytic domain of PCSK9 that prevent its interaction with the LDLR is emphasized. We also show the CHRD modules M1, M2, and M3 and show that sdAb (92, 93) or mAb (94, 95) can block PCSK9 function by binding to the M1 and M3 domains and prevent its sorting to lysosomes. We hypothesize that the interaction of a putative “protein X” with one or more of these modules facilitates the PCSK9-induced degradation of the LDLR.

Binding of PCSK9 to the cell surface LDLR (79, 86, 87, 88) is followed by the internalization of the PCSK9-LDLR complex into clathrin-coated acidic endosomes (89, 90, 91). This stable complex (Fig. 4) is then directed toward endosomes/lysosomes for degradation by an undefined mechanism(s), preventing LDLR recycling to the cell surface (44, 88, 89, 90). Another milestone was achieved when it was shown that such lysosomal targeting pathway requires the participation of the C-terminal Cys-His-rich domain (CHRD) of PCSK9 (89, 92, 94, 96, 97, 98). The latter is composed of three tandem repeats tightly packed into structurally similar modules named M1 (amino acids 453–529), M2 (amino acids 530–603), and M3 (amino acids 604–692) (61, 81). We originally proposed that a yet undefined “protein X” binds the CHRD and directs the PCSK9-LDLR complex to lysosomal degradation (99, 100) (Fig. 4). Interference with this pathway was achieved using single domain antibodies/nanobodies (92, 93) and mAbs (94, 95) that target the M1 and/or M3 modules of the CHRD and inhibit the PCSK9-induced LDLR degradation, without blocking the binding of PCSK9 to the LDLR (Fig. 4). The possible implication of the soluble cytosolic adenyl cyclase-associated protein 1 as a candidate “protein X” has been recently suggested (101). However, the complexity of this sorting mechanism, its enhancement by specific Ser-phosphorylations in the CHRD (102), and the identification of all components of the “protein X” complex required for the trafficking of the PCSK9-LDLR complex to lysosomes are yet to be unraveled in detail.

## Inhibitors of PCSK9 function or synthesis lower LDL-C

It became apparent that the source of circulating PCSK9 originates from hepatocytes and that single LDLR or double LDLR/PCSK9 KO mice exhibited similar cholesterol profiles, indicating that PCSK9 regulates LDL-C exclusively through the LDLR (48). In addition, liver-specific PCSK9 KO exhibited ∼two-thirds of the hypocholesterolemia phenotype observed in complete KO mice (48). This suggested that targeting either circulating or liver PCSK9 should markedly reduce LDL-C and associated atherosclerosis. This conclusion was supported by studies that showed that in mice the absence of PCSK9 substantially reduces the development of atherosclerosis (103), apoB levels, and endothelial dysfunction (104) and that its overexpression does the reverse (103, 105, 106). Since kinetic studies in healthy subjects using an mAb against human PCSK9 do not seem to alter apoB secretion, by rather LDL catabolism (107, 108), whether the effect of PCSK9 overexpression in mice on ApoB secretion is due to an intrahepatic function of PCSK9 is not clear.

**Fig. 5:**
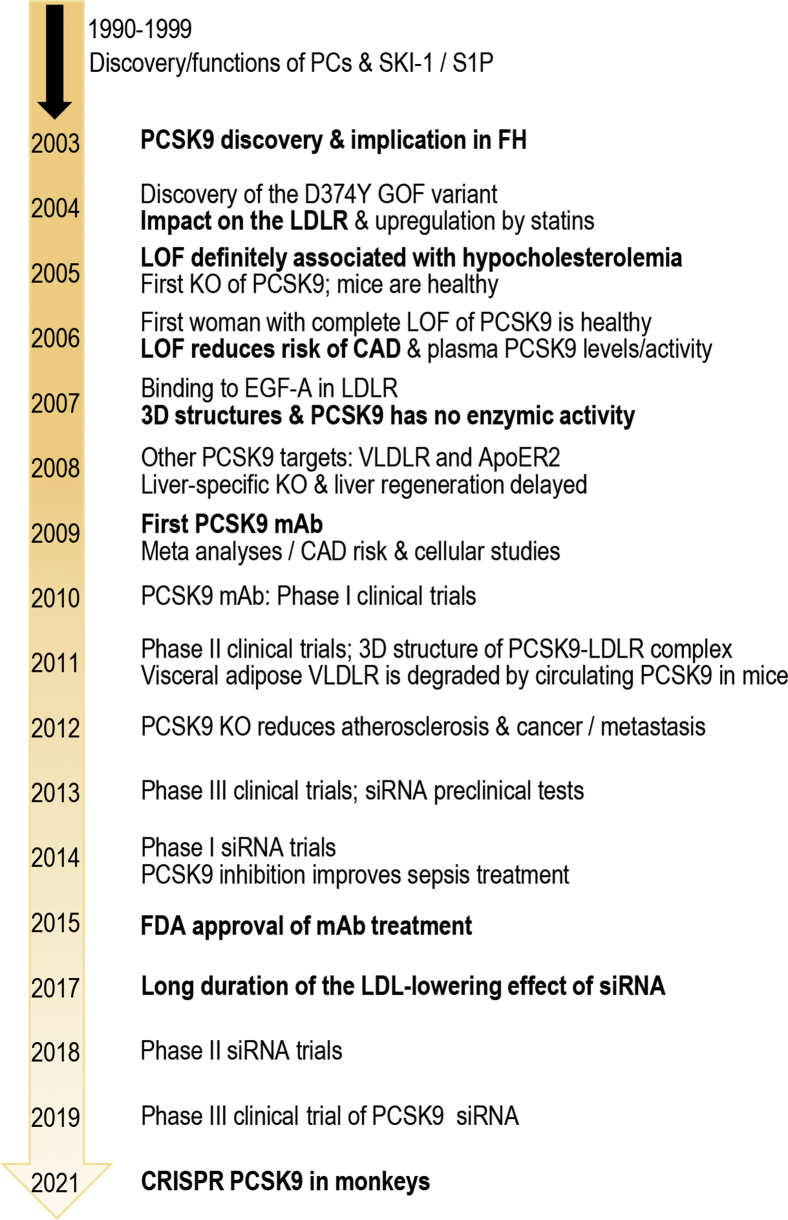
Time line of the critical discovery of PCSK9, its biological functions, the clinical use of either mAb or siRNA treatments, and the preclinical evaluation of a CRISPR approach to delete the gene. The first seven basic amino acid-specific PCs and SKI-1/S1P were identified and characterized during the period of 1990–1999. The complete sequence of human, rat, and mouse PCSK9 was first reported in 2003, as well as the association of the two GOF variants S127R and F216L with FH. The various time lines for the definition of PCSK9 activity and mechanism of function in cells, mouse models, and human ultimately led to the development of potent and safe inhibitory mAb (evolocumab/Repatha and alirocumab/Praluent) following various clinical trials, culminating in 2015–2016 with the Food and Drug Administration approval of the first mAb for clinical use in hypercholesterolemia treatments to reduce coronary artery disease (CAD). The use of liver-targeted nanoparticle carrying a PCSK9 siRNA (inclisiran) received marketing authorization in the European Union in December 2020 and should soon be ready for approval by the Food and Drug Administration. Preclinical evaluations of CRISPR editing of *PCSK9* in monkeys were reported (114).

The aforementioned compelling cellular and genetic evidence of the lipid regulating roles of PCSK9 was extensively reviewed (85, 109, 110, 111) and clearly showed that the absence of PCSK9 activity or mRNA resulted in greatly reduced LDL-C levels in both human and rodents. Such early evidence of the role of PCSK9 in cholesterol regulation led to the rapid development of inhibitory mAb to PCSK9 that blocked its function on the LDLR (112, 113). Optimized versions of these mAbs that underwent extensive clinical trials (evolocumab/Repatha and alirocumab/Praluent) are presently prescribed to patients suffering from hypercholesterolemia in many countries since 2015 (Fig. 5). These subcutaneously injected mAb (biweekly or monthly) reduced LDL-C by ∼50–60% above the level achieved by statins alone (112, 115, 116).

Since loss of PCSK9 expression in liver resulted in substantial reduction of LDL-C, without apparent negative consequences in mice (48), silencing of its hepatic expression was next tested using an injectable lipid nanoparticle formulation carrying an siRNA to PCSK9 (117). Conjugation of antisense oligonucleotides to *N*-acetylgalactosamine mediated their efficient uptake into liver hepatocytes *via* binding to the hepatocyte-specific asialoglycoprotein receptor 1 (118). Accordingly, an optimized siRNA version (inclisiran) is composed of 21 sense and 23 antisense oligonucleotide sequences that have been modified for durability and low immunogenicity. The sense strand is conjugated with triantennary *N*-acetylgalactosamine to facilitate uptake by hepatocytes, thereby enhancing the level and efficacy of the antisense strand liver-specific delivery in order to silence PCSK9 expression. Subcutaneously injected inclisiran two times per year substantially reduces by 50–60% the levels of LDL-C (119, 120).

So far, both mAb (evolocumab and alirocumab) and siRNA (inclisiran) approaches to inhibit PCSK9 function and reduce LDL-C seem safe following 2–5 years of clinical use, but we will have to await longer treatments for a more thorough assessment of the long-term effects of reducing liver *versus* circulating PCSK9. In addition, several other strategies have been proposed to reduce PCSK9 activity or levels (121), including vaccination (122) and clustered regularly interspaced short palindromic repeats base editing to prevent transcription of the full-length PCSK9 gene (114) (Fig. 5). However, the safety of these irreversible approaches to silence PCSK9 function would need long-term evaluations of their effects on many subjects before they can be safely administered on a large scale to humans.

## Inhibitors of PCSK9 in viral infections and cancer/metastasis

The PCs, especially furin and SKI-1/S1P, play major roles in the activation of a number of enveloped viruses (25), including dengue virus (DENV). The latter is a single positive-stranded RNA virus of the family *Flaviviridae*, transmitted to human by the urban-adapted *Aedes* mosquitoes, which yearly infects >400 million individuals worldwide, resulting in ∼25,000 death/year mostly in children from southeast Asia (123). Recently, we showed that in cell cultures, DENV infection enhances the mRNA expression of PCSK9 in hepatocytes (124), thereby reducing cell surface levels of LDLR and LDL-C uptake, resulting in enhanced *de novo* cholesterol synthesis by the SREBP-2 pathway (125). DENV exploits this mechanism for viral packaging. This observation was supported by the detection of elevated plasma PCSK9 levels in patients infected with DENV resulting in high levels of viremia (124). This unexpected role of PCSK9 in dengue pathogenesis led us to test the effect of blocking PCSK9 function by the inhibitory PCSK9-mAb alirocumab. Befittingly, this treatment resulted in higher LDLR levels and lower viremia. More extensive clinical studies are now needed to support a role of PCSK9 inhibition in the treatment of DENV infections and to evaluate the long-term antiviral effect of such treatment.

Cholesterol plays a key role in a plethora of cellular metabolic processes, particularly for highly demanding anabolic steps such as cell growth and division, which are especially relevant to tumor growth and metastasis (126). Indeed, it was reported that PCSK9 deficiency reduces melanoma metastasis in liver (127), and that PCSK9 enhances metastasis of melanoma-derived cells into lung epithelial cells (128). The roles of PCSK9 in cancer have also been highlighted in terms of regulation of inflammation *via* a suppressor of cytokine signaling-3-signal transducer and activator of transcription 3 pathway (129), as well as of cell proliferation and apoptosis (130). In addition, LOF and GOF variants of PCSK9 were associated with lower and higher incidence of breast cancer, respectively (131). Accordingly, it was suggested that PCSK9 expression could be a valuable biomarker for the clinical prognostic outcome of certain types of malignancies, including hepatocellular carcinoma, gastric, kidney, pancreas, and breast cancers (132). However, it is possible that these processes could be related to the PCSK9-induced reduction in the levels of cell surface LDLR and/or other receptors and not only because of its ability to enhance circulating LDL-C levels (133).

In that context, PCSK9 was reported early on to be well expressed in spleen and thymus (41, 85), but its function(s) in these regulatory immune tissues was unknown. Two recent reports shed a completely new light on the function of PCSK9 in the regulation of T-cell activation. It was thus shown that in CD8^+^ T cells, the LDLR heterodimerizes with the T-cell receptor, which is activated upon binding antigenic peptides presented by the major histocompatibility complex (MHC). Thus, reducing circulating PCSK9 (e.g., by an mAb or an siRNA) would increase the levels of the LDLR in CD8^+^ T cells, enhance T-cell receptor recycling and signaling, as well as CD8+ T-cell antitumor activity (134). Independently from the LDLR, PCSK9 *via* the M2 module of its CHRD was also reported to bind MHC-I receptors and to target them to endosomes/lysosomes for degradation (135). Injection of a PCSK9 mAb enhanced the levels of MHC-I receptors and hence T-cell response to several tumors. Furthermore, an mAb against programmed death 1, an immune checkpoint inhibitor widely used in immunotherapy (136), synergized with a PCSK9 mAb in various mouse tumor models to further suppress tumor growth, indicating that inhibition of an LOF of PCSK9 can overcome tumor resistance to anti-programmed death 1 therapy. These unexpected roles for PCSK9, aside from its canonical LDLR regulation, reveal that its functions within the immune system deserve much more detailed and thorough investigations.

Altogether, the use of a safe PCSK9 inhibitor strategy (mAb or siRNA) in combination with statins and possibly ezetimibe should drastically reduce circulating LDL-C levels (137), while enhancing the levels of cell surface LDLR and MHC-I to possibly provide some protection against tumor growth and/or metastasis. Pairing this approach with chemotherapy/immunotherapy has been proposed (138).

## Discussion and conclusions

The PCSK9 discovery in 2003 has extended our understanding of cholesterol metabolism and how LDLRs are upregulated by statins and their protein levels reduced by PCSK9, leading to the design of efficacious strategies to reduce LDL-C for the treatment of hypercholesterolemia and its associated cardiovascular complications. Starting from curiosity-driven and fundamental biology approaches used to identify a new PC, PCSK9 has defied the dogma that PCs require a specific protease activity for their modulation of various secretory substrates. In this case, the protease-dead prodomain-PCSK9 protein complex drags cell surface receptors, such as LDLR, VLDL receptor, ApoER2, LRP1, CD36, and MHC-I to acidic endosomes/lysosomes for degradation (85). Recently, the ER-localized inactive protease zymogen of another convertase PC7 (proPC7; gene *PCSK7*) was also shown to enhance the degradation of apoA-V *via* increased ER-phagy, thereby regulating hepatic triglycerides (139). It would be fascinating if nonenzymatic functions of other PCs can be identified in the future.

Because of the limited repertoire of proteins targeted by PCSK9 for degradation and its high expression in hepatocytes, the inhibition of its function or expression in human patients by mAb or liver-targeted siRNA or even other approaches (121) rapidly became a bench-to-bedside reality in 12–18 years since its discovery (Fig. 5). A multitude of clinical studies clearly demonstrated the high efficacy of PCSK9 inhibitors combined with statins in the treatment of both heterozygote and homozygote FH with residual LDLR activity and in the prevention of atherosclerosis in a wide variety of patients (140, 141, 142). Undoubtedly, new technologies may fuel cheaper and long-lasting PCSK9 inhibitors that would then be more widely prescribed in clinics worldwide. While the physiological functions of PCSK9 in tissues other than liver (143, 144), such as small intestine (145, 146) and pancreatic β-cells (47, 147), are starting to be defined, those in kidney, thymus, brain, and testis (41) still require more investigations. Tissue-specific PCSK9 KO in mouse small intestine (146), pancreatic β-cells (47), and lung epithelia (128) allowed some dissection of the relationship between the autocrine and endocrine functions of PCSK9 but clearly emphasized the dominant role of circulating PCSK9 originating from liver (48, 80). Furthermore, the intracellular activity of PCSK9 found in cell lines (73) seems distinct from that of circulating PCSK9 (148, 149) and may play important roles during embryonic development (41) and in placenta (85, 150), and possibly explain the GOF of some PCSK9 variants.

**Fig. 6:**
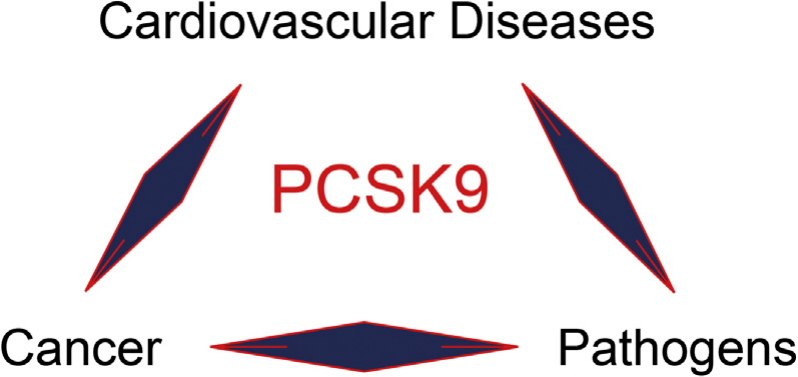
Implication of PCSK9 in at least three major pathologies. Through the regulation of LDLR levels and circulating LDL-C, PCSK9 was convincingly demonstrated to be a major protein regulating hypercholesterolemia and associated cardiovascular diseases. More recently, inhibition of PCSK9 was also shown to attenuate some viral and pathogenic infections such as DENV and those implicated in the development of sepsis. Finally, the ability of PCSK9 to regulate cholesterol as well as to affect the function of the T-cell and MHC-I receptors make it a novel target for the treatment of cancer and its associated metastasis, possibly in combination with immunotherapies and/or chemotherapies. The future will tell if other PCSK9-related functions will be also discovered.

Finally, the future will tell what other functions of PCSK9 could be targeted in pathologies distinct from hypercholesterolemia, such as in cancer/metastasis, viral infections (Fig. 6), and possibly uncontrolled inflammatory or immune reactions, for example, in sepsis (151) implicating pathogen lipid clearance *via* the LDLR (152, 153, 154, 155), in vascular inflammation (156), and in platelet activation (157).

## Data availability

There are no new data to disclose.

## Conflict of interest

The author declares no conflicts of interest with the contents of this article.
